# Rare Condition of Dens Invaginatus in a Maxillary Primary Molar and a Birooted Maxillary Primary Canine diagnosed during Routine Examination

**DOI:** 10.5005/jp-journals-10005-1433

**Published:** 2017-06-01

**Authors:** Eliana Costa Barbosa Brandão, Apoena Aguiar Ribeiro, Luciane Monte Alto Seabra

**Affiliations:** 1 Private Practitioner, Department of Pediatric Dentistry, Estacio de Sa University Rio de Janeiro, Brazil; 2 Associate Professor, Department of Pediatric Dentistry and Cariology, Fluminense Federal University, Rio de Janeiro, Brazil; 3 Associate Professor, Department of Pediatric Dentistry, Estacio de Sa University Rio de Janeiro, Brazil

**Keywords:** Birooted teeth, Dens invaginatus, Tooth morphology.

## Abstract

Congenital morphological dental alterations are related to the physiological phase of tooth development. Since the etiology of the dens invaginatus and supernumerary roots has yet to be fully determined, a rare presentation of dens invaginatus in a primary molar and a birooted deciduous maxillary canine were incidentally detected in routine radiographic examinations. Essential clinical considerations and treatment options are presented.

**Clinical significance:**

This study highlights the importance of a deep and comprehensive anamnesis and clinical examination, with radiographs taken when necessary. The negligence of these stages in an initial consultation could cause irreversible future damage to the patient, since rare dental anomalies or other lesions that require treatment may not be detected.

**How to cite this article:**

Brandão ECB, Ribeiro AAA, Seabra LMA. Rare Condition of Dens Invaginatus in a Maxillary Primary Molar and a Birooted Maxillary Primary Canine diagnosed during Routine Examination. Int J Clin Pediatr Dent 2017;10(2): 193-195.

## INTRODUCTION

Tooth development or odontogenesis initiates in the ectoderm and mesoderm of the primitive oral cavity. The ectoderm originates the enamel organ, i.e., composed of the outer enamel epithelium, inner enamel epithelium, stellate reticulum, and stratum intermedium. Cells from inner enamel epithelium give rise to ameloblasts which produce enamel, whereas the mesoderm produces the dental papilla which differentiates in odontoblasts to form dentin and dental pulp. Cementoblasts, osteoclasts, and fibroblasts are formed from the dental sac. Congenital dental alterations are related to the physiological stage of the development of the tooth, allowing us to identify the stage at which they developed.^1^

Dens invaginatus, also known as dens in dente and dilated compound odontoma, is a malformation that can occur on primary, permanent, or supernumerary teeth. It is characterized by a deep invagination of the surface of a crown or root, covered with enamel. This alteration is related to the proliferation and morphodifferentiation phase. Controversy exists regarding the etiology of this morphological alteration, although it probably occurs as a result of an extra invagination of the inner enamel epithelium in the interior of the tooth crown before its mineralization, presenting as consequence, an enamel and dentin intra-crown formation.^1,2^

The first report of a dens invaginatus is from 1952, when Rabinowitch reported a case of a 3-year-old white boy showing a dens in dente in the upper left primary molar.^3^ A review of the literature indicates that dens invaginatus is extremely rare in primary teeth, with only four cases being reported until nowadays.^1,4-6^

Clinically, diagnosis of this anomaly is extremely important since the site of the invagination predisposes the tooth to caries development due to the difficulty in access for cleaning, possibly leading to pulp necrosis, periapical lesions, or periapical abscesses. The complex morphology of these cases makes the treatment of the root channels more complicated. However, endodontic treatment of dens invaginatus may be conventional and in some cases, associated to a retro obturation.

The extranumerary roots are associated with the root phase formation during the physiological stages of tooth formation.^1^ Barker,^7^ in his study of dental anthropology, mentioned that the finding of permanent inferior canines with forked roots is common. However, birooting in deciduous canines is a rare condition with unknown etiology. According to Kelly,^8^ this anomaly occurs as a result of an internal growth of the Hertwig’s root sheat, although relatively few reports regarding this condition may be found in the literature.^9-14^

This report describes two rare dental morphological alterations, dens invaginatus and a birooted deciduous canine, diagnosed in the same patient during a radio-graphical examination in a routine appointment.

**Fig. 1:**
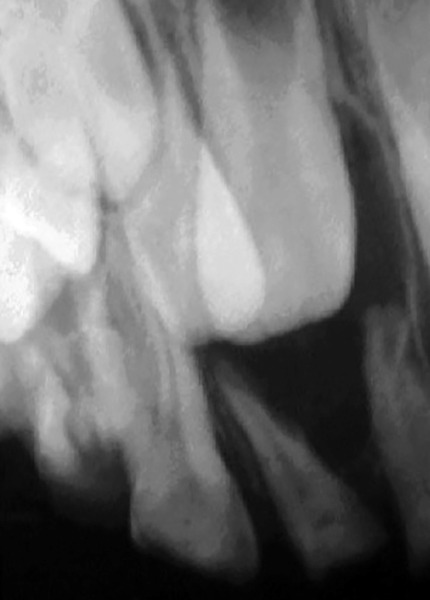
Periapical radiograph showing primary maxillary right birooted canine

**Fig. 2:**
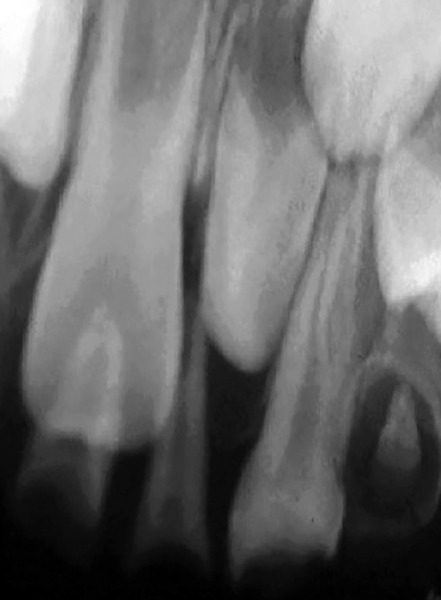
Periapical radiograph showing dens invaginatus in a primary maxillary left first molar

## CASE REPORT

A 5-year-old male of African ascendancy was attended at the pediatric dentistry clinic from a private Brazilian university. After parent signed the consent form, anamnesis and routine clinical examination was conducted. Patient’s medical history did not relate to any unusual occurrences, however, during clinical examination, extensive dental crown destruction by dental caries was observed in all upper elements, in the four lower deciduous molars and on the vestibular face of the inferior primary canine. A routine bitewing radiograph identified an uncommon pulp anatomy in the superior deciduous canine, suggesting a bifurcation of its roots. This diagnosis was confirmed by periapical radiograph (Fig. 1). In addition, the presence of dens invaginatus was also observed in the upper left primary first molar, reaching the pulp chamber (Fig. 2).

Initially, provisory restorations were made with glass ionomer cement and instructions for oral hygiene and diet control were given. Esthetical reconstruction with composite was proposed for the superior deciduous canines and the first left superior deciduous molar was extracted due to advanced inflammatory pulp involvement, with a space maintainer installation. The other elements were restored for reestablishment of function and esthetics.

## DISCUSSION

The initial stages of dental examinations are extremely relevant for successful diagnosis, prevention, and treatment of oral pathologies. Anamnesis and clinical examination should be accomplished in all situations, with radiographs taken when necessary. The negligence of these stages, in an initial consultation, could cause irreversible future damage to the patient, since dental anomalies or other lesions that require treatment may not be detected.

In a review of the literature, Pindborg^15^ registered a prevalence of dens invaginatus ranging from 0.25 to 5.1%, affecting maxillary lateral incisors among several population groups. Following maxillary lateral incisors in decreasing order of frequency are: Central incisors, premolars, canines, and the last molars.^16^ Instances of dens invaginatus affecting primary teeth have also been reported although these are uncommon.^3-6^

In this report, two morphological dental alterations were diagnosed in the primary teeth. These alterations would not have been observed without radiographic examinations. Thus, it should be emphasized that radiographs are an important complementary exam for correct diagnosis and should always be initially accomplished for the elaboration of a correct treatment plan.

According to Hulsmann and Hengen,^17^ dens invaginatus increases predisposition to the installation of decay lesions. In this case, we were unable to detect whether the presence of this anomaly facilitated the development of disease, because when the patient was seen by the pediatric dentistry, the dental crown was already completely destroyed and pulp vitality was compromised, making its removal necessary. Since the other elements also presented extensive carious lesions, we suggest that this morphological alteration was probably not the main cause of the installation of disease, but just one more unrelated lesion factor.

Some authors have observed the presence of birooted deciduous canines during routine radiographic examinations. This is an asymtomatic morphologic alteration that does not interfere with the normal development of the primary and permanent teeth.^9-14^ However, only four reports describing dens invaginatus in deciduous molars were found in the literature, suggesting that this case is a rare case of morphologic alteration in primary teeth. Moreover, no case report was found with both conditions, suggesting that this case is the first to be registered.

## CONCLUSION

It is very important that all the initial stages of dental treatment consultations should be accomplished, so that, no abnormality of morphological character (or otherwise) be neglected. A detailed anamnesis is fundamental at the first consultation in addition to a rigorous clinical examination and the solicitation of radiographic and complementary exams. In this report, the radiography was of fundamental importance for the observation, diagnosis, and treatment of the dental anomalies found.

## CLINICAL SIGNIFICANCE

This study highlights the importance of a deep and comprehensive anamnesis and clinical examination, with radiographs taken when necessary. The negligence of these stages, in an initial consultation, could cause irreversible future damage to the patient, since rare dental anomalies or other lesions that require treatment may not be detected.
